# Chimaeric Virus-Like Particles Derived from Consensus Genome Sequences of Human Rotavirus Strains Co-Circulating in Africa

**DOI:** 10.1371/journal.pone.0105167

**Published:** 2014-09-30

**Authors:** Khuzwayo C. Jere, Hester G. O'Neill, A. Christiaan Potgieter, Alberdina A. van Dijk

**Affiliations:** 1 Biochemistry, Centre of Human Metabonomics, North-West University, Potchefstroom, South Africa; 2 Institute of Infection and Global Health, University of Liverpool, Liverpool, United Kingdom; 3 Department of Microbiology, Biochemistry and Food Biotechnology, University of the Free State, Bloemfontein, South Africa; 4 Deltamune (Pty.) Ltd., Lyttelton, Centurion, South Africa; The Pirbright Institute, United Kingdom

## Abstract

Rotavirus virus-like particles (RV-VLPs) are potential alternative non-live vaccine candidates due to their high immunogenicity. They mimic the natural conformation of native viral proteins but cannot replicate because they do not contain genomic material which makes them safe. To date, most RV-VLPs have been derived from cell culture adapted strains or common G1 and G3 rotaviruses that have been circulating in communities for some time. In this study, chimaeric RV-VLPs were generated from the consensus sequences of African rotaviruses (G2, G8, G9 or G12 strains associated with either P[4], P[6] or P[8] genotypes) characterised directly from human stool samples without prior adaptation of the wild type strains to cell culture. Codon-optimised sequences for insect cell expression of genome segments 2 (VP2), 4 (VP4), 6 (VP6) and 9 (VP7) were cloned into a modified pFASTBAC vector, which allowed simultaneous expression of up to four genes using the Bac-to-Bac Baculovirus Expression System (BEVS; Invitrogen). Several combinations of the genome segments originating from different field strains were cloned to produce double-layered RV-VLPs (dRV-VLP; VP2/6), triple-layered RV-VLPs (tRV-VLP; VP2/6/7 or VP2/6/7/4) and chimaeric tRV-VLPs. The RV-VLPs were produced by infecting *Spodoptera frugiperda* 9 and *Trichoplusia ni* cells with recombinant baculoviruses using multi-cistronic, dual co-infection and stepwise-infection expression strategies. The size and morphology of the RV-VLPs, as determined by transmission electron microscopy, revealed successful production of RV-VLPs. The novel approach of producing tRV-VLPs, by using the consensus insect cell codon-optimised nucleotide sequence derived from dsRNA extracted directly from clinical specimens, should speed-up vaccine research and development by by-passing the need to adapt rotaviruses to cell culture. Other problems associated with cell culture adaptation, such as possible changes in epitopes, can also be circumvented. Thus, it is now possible to generate tRV-VLPs for evaluation as non-live vaccine candidates for any human or animal field rotavirus strain.

## Introduction

Human rotaviruses are the main cause of severe infant gastroenteritis. Rotavirus disease is associated with approximately 453 000 annual childhood deaths of which most occur in developing countries [1]. The use of rotavirus vaccines at an early age is the first line of defence against severe rotavirus disease. RotaTeq and Rotarix vaccines were recommended by the WHO for routine use in children across the globe [2], [3]. Although these vaccines have been shown to be effective in preventing severe rotavirus disease [4], [5], their use has revealed some shortcomings. Their high cost is beyond reach of most developing nations. The lower efficacy of these vaccines in developing countries [6]–[9] compared to their efficacy in developed countries [6] and reassortment with wild-type strains during mixed infections [10], which is common in developing countries [11], is another cause for concern. Some studies suggest that neutralizing activity of immunoglobulin A [12] and the synergistic inhibitory effects of non-antibody components present in breast milk [13] could also compromise the oral rotavirus live-attenuated vaccine take. Furthermore, the current commercial producers of rotavirus vaccines would not meet the global demand if all countries were to introduce mandatory rotavirus vaccination in all infants [14]. RotaTeq [from strains WC3, WI78, WI79, SC2, BrB [15]] and Rotarix [from strain 89–12 [16]] were formulated from strains circulating in USA between 1981 and 1998. Rotarix was adopted under the assumption that it would render cross-reactive antibody protection whereas the VP4 and VP7 of RotaTeq represented the most prevalent serotypes of the strains that were circulating at that time. Since then, wide strain diversity have been reported particularly in developing countries [11], [17]. There is thus a need for further development of alternative rotavirus vaccine candidates and strategies.

Amongst others, RV-VLPs are some of the promising candidates that are currently considered as a potential viable alternative option [18], [19]. This is based on the fact that RV-VLPs are (i) non-infectious as they do not contain genomic material and thus cannot replicate [19]; (ii) are highly immunogenic when formulated with appropriate adjuvants [20] as the viral proteins are in their natural conformation [21]; (iii) can be genetically manipulated to provide broader protection by incorporating several rotavirus serotypes [19], [22]; and (iv) are amenable to large-scale production [23]. RV-VLPs have also been used in basic research to understand the structural conformation of rotavirus particles, functions of the structural proteins and understanding the interaction between rotaviruses and their hosts [19], [24]–[26]. In nano-technology, RV-VLPs can be employed as possible drug delivery systems to the gut as novel nano-carriers due to their natural epithelial cell tropism that can efficiently transfer bioactive molecules to other specific target tissues [27]. However, most RV-VLPs have been prepared for the purpose of developing rotavirus vaccine candidates [19], [20].

*Rotavirus* represents a genus in the *Reoviridae* virus family. It has a dsRNA genome consisting of 11 segments that encodes six structural (VP1–VP4, VP6 and VP7) and six nonstructural (NSP1–NSP6) viral proteins. The three capsid protein layers that enclose the genome are composed of 60 dimers of the 102 kDa VP2, inner capsid [25]; 260 trimers of the 45 kDa VP6, middle layer capsid [28]; and 780 monomers (260 trimers) of the 37 kDa VP7 and spikes formed by 60 trimers of the 87 kDa VP4, outer capsid [28], [29], [30]. The commonly used dual rotavirus classification system (G and P) is based on the properties of the two outer capsid proteins, VP4 (protease sensitive) and VP7 (a glycoprotein) [31]. To date, 37 P and 27 G rotavirus genotypes have been defined and different genotypes have been described for the other 9 genome segments [32]–[34], illustrating the diversity of rotavirus strains.

Single-layered VP2 (sRV-VLPs), double-layered VP2/6 (dRV-VLPs), and triple-layered VP2/6/7 or VP2/6/7/4 (tRV-VLPs) RV-VLPs have previously been produced successfully in baculovirus vector expression systems (BVES) by using either mono-cistronic [22], [35] or multi-cistronic [36] expression strategies. Co-infection strategies make use of either mono-cistronic or dual-cistronic baculoviruses expressing either a single or two rotavirus proteins, respectively. To date, there are no data on tRV-VLPs formulated from human rotavirus strains that emerged in the past two decades. All tRV-VLPs have been generated from cell-culture adapted rotavirus strains. Adaptation of wild-type human rotaviruses to cell culture requires specialised skilled personnel, is resource demanding and also very time-consuming [37]–[39]. Adapting rotavirus strains to cell culture may result in changes especially to VP4 binding regions that are involved in viral attachment to host cellular receptors such as glycans and A-type histo-blood group antigens [40], [41]. In addition, rotaviruses exist as populations of quasiespecies of different composition whereby environmental and host factors dictate which population persists [42]. Cell culture adapted strains may undergo as yet unknown genetic drift resulting in biological changes that enable a proportion of the quasiespecies population to adapt to the artificial *in vitro* environment which may cause them to have phenotypic differences to wild-type strains. Therefore, preparing RV-VLPs directly from field strains using the consensus expression system-based codon-optimised sequences derived from their dsRNA may represent a novel strategy of producing non-live RV-VLP vaccines from any human or animal field strains without the prior intermediate step of cell culture adaptation. RV-VLPs produced in this manner may not only shorten the time required to prepare RV-VLPs from wild-type strains, but also may permit production of RV-VLPs with antigenic characteristics that closely mimic the current circulating wild-type strains. In the current study, the possibility of generating chimaeric RV-VLPs using a common DS-1-like VP2/6 dRV-VLP backbone was investigated. Outer capsid proteins of selected prevalent rotavirus field strains in Africa and those that emerged in the past two decades of which their dsRNA was isolated directly from human stool samples were used.

## Materials and Methods

### 2.1. Rotavirus strains

Species A human rotavirus strains (RVA) of African origin characterised directly from stool samples (Table 1) were used to prepare chimaeric RV-VLPs. The extraction of rotavirus dsRNA, cDNA synthesis, sequence-independent whole genome amplification, 454 pyrosequencing and determination of consensus sequences used in the current study is detailed in the literature [43]–[45]. RVA strains containing either a G2, G8, G9 or G12 VP7 encoding genome segment 9 associated with either P[4], P[6] or P[8] VP4 encoding genome segment 4 were solicited from the stool sample collections of the National Institute for Communicable Diseases (NICD), Johannesburg, South Africa and the WHO Regional Reference Laboratory/Diarrhoeal Pathogens Research Unit (DPRU), Pretoria, South Africa. The genotypes of these strains have been either detected frequently in some areas where vaccines have been introduced (G2s in Australia and Brazil) [46], [47], frequent detection in Africa (G8s) or have emerged in the past two decades (G9s and G12s) [11], [48], [49].

**Table 1:** Rotavirus strains and restriction endonucleases used to clone selected VP4 and VP7 encoding ORFs into the pFBq donor plasmid.

Strain	GenBank Accession number	Encoded protein (Genotype)	Restriction enzyme	Promoter^a^
RVA/Human-wt/ZAF/3203WC/2009/G2P[4]	HQ657176	VP7 (G2)	*Bam* HI and *Not* I	polh
RVA/Human-wt/MWI/1473/2001/G8P[4]	HQ657143	VP7 (G8)	*Bam* HI and *Not* I	polh
RVA/Human-wt/ZAF/GR10924/1999/G9P[6]	FJ183360	VP7 (G9)	*Bam* HI and *Not* I	polh
RVA/Human-wt/ZAF/3176WC/2009/G12P[6]	HQ657165	VP7 (G12)	*Bam* HI and *Not* I	polh
RVA/Human-wt/ZAF/3133WC/2009/G12P[4]	HQ657174	VP4 (P[4])	*Spe* I and *Sma* I	p10
RVA/Human-wt/ZAF/GR10924/1999/G9P[6]	HQ657152	VP4 (P[6])	*Spe* I and *Sma* I	p10
RVA/Human-wt/ZAF/2371WC/2009/G9P[8]	JN013994	VP4 (P[8])	*Spe* I and *Sma* I	p10
RVA/Human-wt/ZAF/GR10924/1999/G9P[6]	FJ183354	VP2 (C2)	EcoRI	p10
RVA/Human-wt/ZAF/GR10924/1999/G9P[6]	FJ183358	VP6 (12)	XbaI	polh
				

a The ORFs coding for the rotavirus proteins were inserted downstream of these promoters as indicated in Fig.1.

### 2.2 Construction of recombinant baculoviruses expressing chimaeric RV-VLPs

The Bac-to-Bac Baculovirus Expression System (BEVS; Invitrogen) was used for the production of chimaeric RV-VLPs according to the manufacturer's specifications with minor deviations. pFastBACquad (pFBq, Fig.S1) was used as a donor plasmid to prepare expression cassettes in which various combinations of open reading frames (ORFs) coding for VP4 and VP7 from different rotavirus strains were cloned. pFBq was generated by incorporating the multiple cloning site (MCS) of pBacgus4x-1 (Novagen, Merck Chemicals Ltd., Nottingham, UK), which allows cloning and co-expression of up to four target genes under the control of two polyhedron (polh) or two p10 *Autographa californica* multi-capsid nucleopolyhedrosis virus (AcMNPV) promoters, into pFastBac (Invitrogen, Life Technologies, Grand Island, NY).

ORFs coding for VP2, VP4, VP6 and VP7 derived from the consensus nucleotide sequences of the selected rotavirus strains (Table 1) were codon-optimised for insect cell expression at GenScript using the OptimumGene algorithm (GenScript USA Inc. New Jersey, NJ) that considers several factors that regulate and influence gene expression levels to ensure the highest possible level of expression in insect cells. The basic principal is to maintain the amino acid sequence of the capsid proteins [50]. All synthetic nucleotide sequences were designed to contain restriction endonuclease (RE) sites at the 5'- and -3' ends (listed in Table 1), flanking the ORFs, to facilitate sub-cloning. Two stop codons were inserted at the -3' end. The synthetic cDNA encoding the ORFs of VP2, VP4, VP6 and VP7 were purchased from GeneArt (Life Technologies, New York, NY) and GenScript (GenScript USA Inc. New Jersey, NJ). The ORFs coding for the rotavirus proteins were inserted downstream of the polh (VP6 and VP7) and p10 (VP2 and VP4) promoters that regulated their expression (Table 1; Fig. 1). Successful cloning of the ORFs into pFBq was verified through Sanger sequencing at the Central Analytical DNA Sequencing Facility (Stellenbosch University, South Africa).

**Figure 1:**
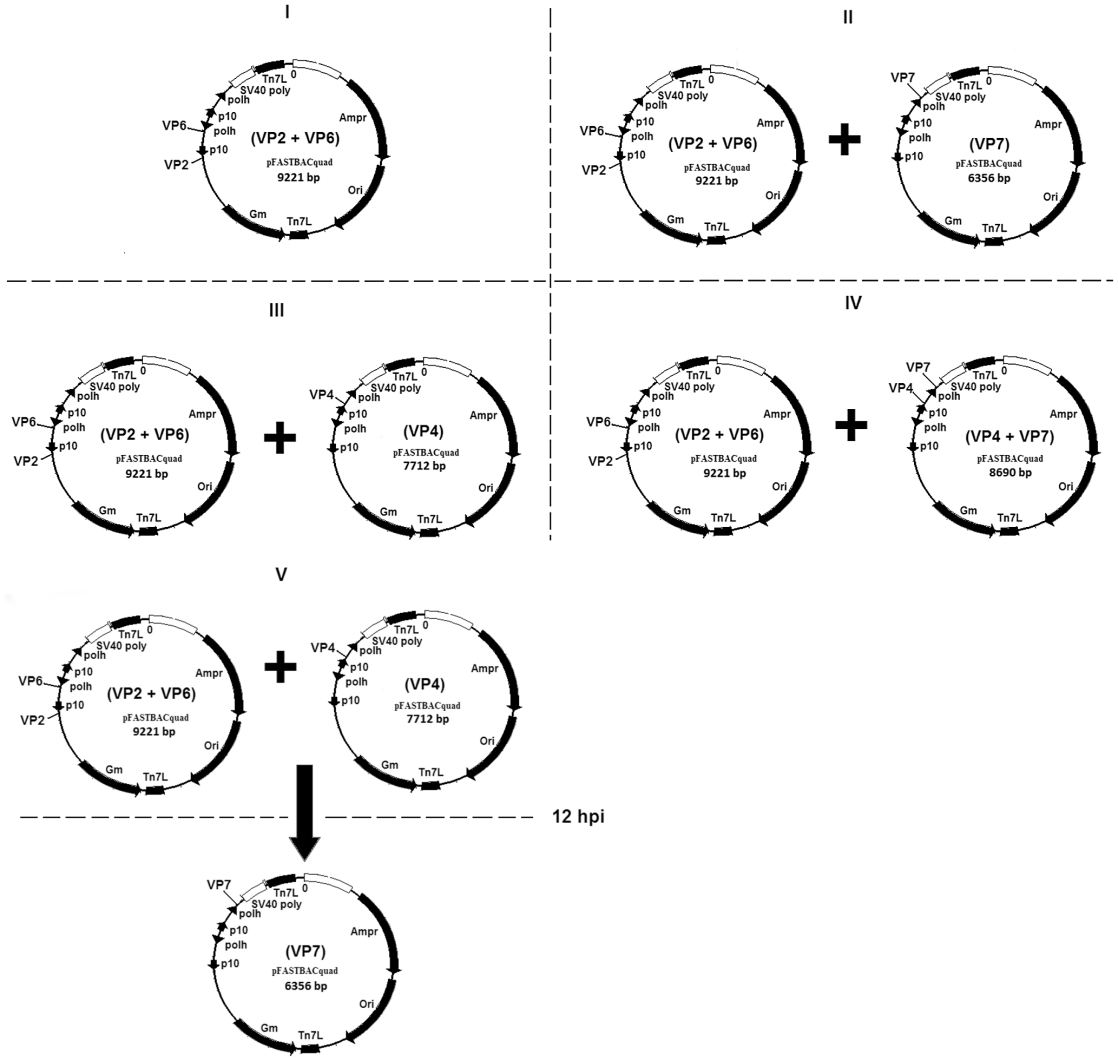
Schematic presentation of the baculovirus expression strategies used to generate RV-VLPs. The donor plasmids contains ORFs coding for specific rotavirus proteins (labelled downstream to the promoters regulating their expression as described in the text) that were transposed into bacmids which were subsequently used to generate baculoviruses. The restriction sites used for construction of the recombinant transfer plasmids are not indicated on the pFBq plasmids maps above, see Fig. S1 and Table 1 for more details. (**I**) pFBq plasmid construct used to generate recombinant dualcistronic baculoviruses that was used to prepare dRV-VLPs (VP2/6) through single infection of insect cells. (**II**) pFBq plasmid constructs used to generate recombinant dualcistronic and monocistronic baculoviruses that were used to prepare tRV-VLPs (**VP2/6/7**) through simultaneous infection of insect cells. (**III**) pFBq plasmid constructs used to generate recombinant dualcistronic and monocistronic baculoviruses that were used to prepare tRV-VLPs (**VP2/6/4**) through simultaneous infection of insect cells. (**IV**) pFBq plasmid constructs used to generate recombinant dualcistronic baculoviruses that were used to prepare tRV-VLPs (**VP2/6/7/4**) through simultaneous infection of insect cells. (**V**) pFBq plasmid constructs used to generate recombinant dualcistronic and monocistronic baculoviruses that were used to prepare tRV-VLPs (**VP2/6/7/4**) through step-wise co-infection strategy. Insect cells were initially infected with dualcistronic baculoviruses confirmed to express VP2/6 and recombinant monocistronic baculoviruses confirmed to express VP4. This was followed by infection with recombinant monocistronic baculoviruses confirmed to express VP7 12 hours post initial infection (hpi).

The *E. coli* strain AcBACΔCC [51] was used to construct and propagate recombinant baculovirus expression shuttle vectors (bacmids). Various pFBq expression cassettes were transformed as described in the BEVS manual into chemically competent AcBACΔCC *E.coli* cells to produce the recombinant bacmids. High molecular weight bacmid DNA was isolated from five colonies per construct using the BACMAX DNA purification kit (Epicentre Biotechnologies, Madison, WI) by following the manufacturer's instructions. Selection of recombinant bacmids were carried out by PCR as described in the BEVS manual using TaKaRa Ex-Taq DNA polymerase (Takara Bio Inc., Shiga, Japan).

*Spodoptera frugiperda* (Sf) 9 cells, maintained in complete TC-100 medium (Sigma-Aldrich, St. Louis, MO) containing 10% foetal bovine serum (FBS), 1% antibiotic (streptomycin/penicillin, Lonza, Wakersville, MD) and 1% fungizone (amphothericin B, Lonza, Wakersville, MD), were transfected with each of the five recombinant bacmid DNAs isolated for each pFBq construct. FuGENE 6 and X-tremeGENE transfection reagents (Roche Diagnostics, Mannheim, Germany) were used per manufacturer's instructions. The 6-well plates were incubated at 28°C until the cytopathic effect (CPE) was complete. The transfection supernatant was harvested from each well, centrifuged at 500×g for 2 min, and the supernatant (P1 viral stock) was stored at 4°C for infection experiments. Plaque purifications were performed and 10 separate plaques per recombinant baculovirus were selected.

### 2.3 Verification of recombinant protein expression

Expression of recombinant rotavirus proteins by the plaque purified baculoviruses was verified by harvesting cell pellets from each virus stock followed by performing SDS-PAGE and western blot analysis. The cell pellet was lysed for 1 h with 200 µl of cell lysis buffer (10 mM Tris (Sigma-Aldrich, Ayrshire, KA), 0.1 mM ethylene-diamine-tetra-acetic acid (EDTA: Sigma-Aldrich, St. Louis, MO), 1% sodium deoxycholate (DOC: Sigma-Aldrich, St. Louis, MO), 0.1% sodium dodecyl sulphate (SDS: Sigma-Aldrich, St. Louis, MO) and complete protease inhibitor cocktail tablets by following manufacturer's instructions (Roche Diagnostics, Mannheim, Germany). 14 µl of the cell lysate was analysed on SDS-PAGE gels which were stained with Coomassie blue [52]. Western blot analysis was used to verify expression of the recombinant proteins, especially VP7. Following transfer of proteins onto nitrocellulose membrane (Whatman GmbH. Dassel, Germany), the membranes were blocked with 5% non-fat skim milk in TNT buffer [0.05% Tween, 0.2 M NaCl and 0.05 M Tris-HCl (pH 7.4)] for 3.5 h at 4°C. The membranes were incubated for 8 h at 4°C with goat polyclonal anti-rotavirus antibody prepared against rotavirus Nebraska calf diarrhoea virus (NCDV) antibody (Biotin) (ab69560) (Abcam, San Francisco, CA) which was diluted to 1∶1000 ratio in TNT buffer followed by incubation in the secondary antibody, donkey horseradish peroxidase conjugated anti-goat IgG (Abcam San Francisco, CA) diluted 1: 500 in TNT buffer for 1 h. The membranes were washed three times with TNT buffer, followed by incubation until bands were visible in 4-chloro-1-naphthol peroxidase substrate prepared by dissolving 1 tablet of 4-chloro-1-naphthol (Sigma-Aldrich, St. Louis, MO) in 10 ml of methanol (Merck, Darmstadt, Germany) as stock solution. Then 2 ml of the methanol stock solution was added to 10 ml PBS, pH 7.5. This was followed by adding 5 µl of fresh 30% hydrogen peroxide (Sigma-Aldrich, St. Louis, MO) immediately prior to use.

### 2.4. Production of chimaeric RV-VLPs and sucrose gradient fraction purification

RV-VLPs were produced in Sf9 or High Five (Invitrogen) cells in shaker cultures. Sf9 cells were maintained in Ex-Cell Titre High Medium (SAFC Biosciences, Lenexa, KS) containing 10% FBS (Gibco, Life Technologies, Grand Island, NY), 1% Penicillin/Streptomycin/Amphotericin B (Lonza, Wakersville, MD), 0.1% Pluronic F-68 solution (Sigma-Aldrich, Ayrshire, KA) and 0.5–1 µg/ml Leupeptin or Aprotinin protease inhibitors (Roche Diagnostics, Mannheim, Germany). High Five cells were maintained in Express 5 serum-free medium (Gibco, Life Technologies, Grand Island, NY) containing 0.6 mg/L L-glutamine (Sigma-Aldrich, Ayrshire, KA), 10 µg/ml gentamycin (Sigma-Aldrich, Ayrshire, KA) and 0.5–1 µg/ml Leupeptin or Aprotinin protease inhibitors (Roche Diagnostics, Mannheim, Germany). The infected cultures were incubated at 28°C and shaking at 96 rpm until CPE reached approximately 95% (between 5–6 days).

During preliminary investigations in our laboratory, MOI of 0.1, 1, 2, 5 and 10 pfu/cell were compared for expression of recombinant rotavirus proteins and dRV-VLP formation. A MOI of 2 pfu/cell was shown to give the best yield of dRV-VLP formation (M.J. van der Westhuizen, personal communication). Therefore, RV-VLPs were produced by infecting either Sf9 or High Five insect cells with one or more baculoviruses containing the ORF (s) encoding one or more rotavirus structural proteins at a MOI of 2 pfu/cell. To produce dRV-VLPs (VP2/6) from the GR10924 G9P[6] strain that was used as a backbone of all RV-VLPs, Sf9 or High Five cells at a density of 1×10^6^ cells/ml in 100 ml shaker cultures were infected with viral stocks confirmed to simultaneously express VP2 and VP6 proteins. Different strategies were used to produce chimaeric RV-VLPs consisting of VP2/6/4, VP2/6/7 and VP2/6/4/7 protein layers, detailed in Fig. 1. A stepwise infection was also followed to allow for proper mounting of VP4 on the dRV-VLPs prior to VP7 assembly as recommended by Trask and Dormitzer [53].

The cells and medium were harvested 5–6 days post-infection (dpi) followed by centrifugation at 3,000×*g* for 15 min at 4°C. The cell pellet was resuspended in 10 ml lysis buffer and centrifuged at 3,000×*g* for 15 min at 4°C to remove cell debris. The supernatant recovered from culture and cells was layered on a 40% sucrose cushion prepared in Tris-calcium buffer (10 mM Tris-HCl [pH 7.4], 10 mM CaCl_2_) followed by ultracentrifugation in a Sorvall WX Ultra Series centrifuge (Thermo Fisher Scientific Inc., Waltham, MA) using a Surespin 630 rotor (36 ml) at 135,172×*g* for 2 h at 4°C. The supernatant was discarded and the pellet was resuspended in 400 µl Tris-calcium buffer of which 100 µl was applied to the top of 10%–60% sucrose gradients prepared in Tris-calcium buffer [54]. The samples were centrifuged for 1 h at 106, 401×*g* in a Sorvall TH-660 rotor (Thermo Fisher Scientific Inc., Waltham, MA) at 4°C. The bands containing RV-VLPs were visualised with the Gradient Station *ip* fractionator (BioComp Instruments Inc, New Brunswick, Canada) which was also used to collect 18 fractions of 220 µl each from sucrose gradients by following the manufacturer's instructions. The protein concentration of the RV-VLPs was determined using a Bicinchoninic Acid (BCA) Protein Assay Reagent (Pierce, Rockford, IL) by following the manufacturer's instructions.

### 2.5. Verification of the production of chimaeric rotavirus-like particles

To verify the production of RV-VLPs, the presence of the recombinant rotavirus structural proteins was determined by screening the gradient fractions using SDS-PAGE or western blot analysis as described in section 2.3. The fractions confirmed to contain RV-VLPs were pooled and washed using Tris-calcium buffer followed by ultracentrifugation at 106,401×*g* for 1 h at 4°C. The pellet was suspended in 100 µl Tris-calcium buffer. The structural integrity of the RV-VLPs was verified by negative staining the samples on a 0.5% Formvar-coated copper grid using 1% uranyl acetate stain (Kim et al., 2002) followed by examination using two transmission electron microscopes (TEM) [A Jeol JEM-1200 Mk-I (JEOL Ltd, Tokyo, Japan) at Onderstepoort Veterinary Institute, South Africa, and Carl Zeiss TEM (Carl Zeiss NTS GmbH, Oberkochen, Germany) at University of Limpopo, Medunsa, South Africa].

## Results

### 3.1. Recombinant rotavirus protein expression in insect cells

**Figure 2:**
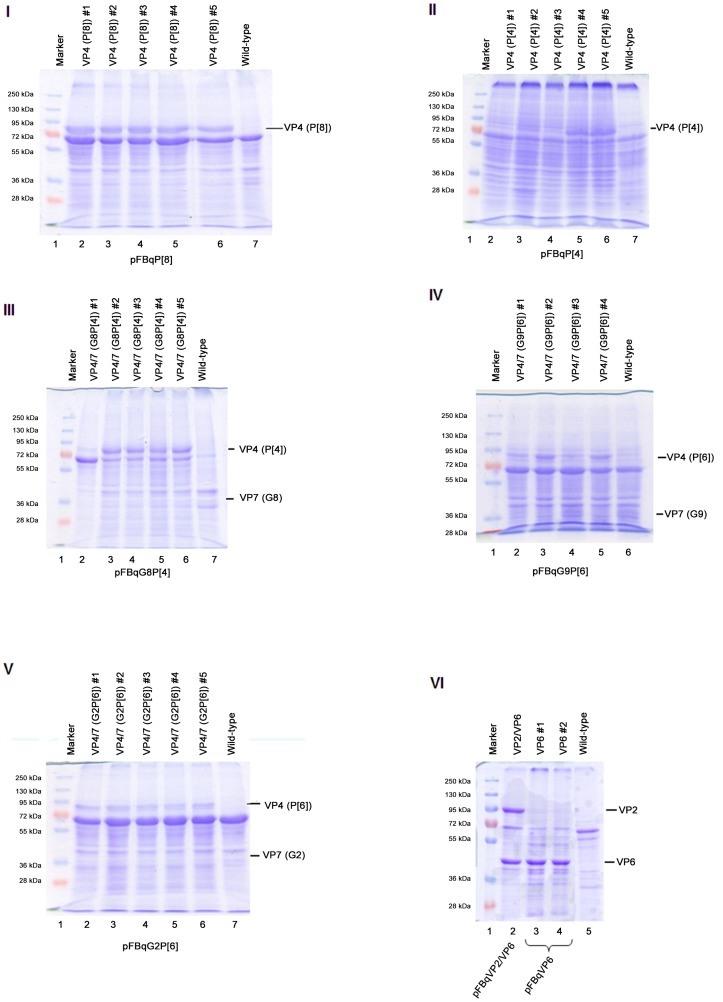
SDS-PAGE gels of recombinant rotavirus proteins expressed by recombinant baculoviruses. (**I** and **II**) Expressed recombinant VP4 with P[4] and P[8] genotypes, respectively. (**III, IV** and **V**) VP4 with P[6] genotypes expressed by recombinant dualcistronic baculoviruses containing ORFs coding for VP4 and VP7. Expressed VP7 was not detected following staining with Coomasie brilliant blue. (**VI**) Recombinant VP2 and VP6 (lane 2) expressed by dualcistronic baculoviruses containing VP2 and VP6 encoding ORFs, and recombinant VP6 (lanes 3 and 4) expressed by monocistronic baculoviruses containing the VP6 encoding ORF. #1, #2, #3, #4 and #5 designate different plaques purified from the same construct on each gel. Lanes: Ladder, PageRuler Plus Prestain Protein Ladder (Fermentas UAB, Vilnius, Lithuania); Wild-type, empty baculovirus used as a control.

Production of recombinant proteins that self-assemble into VLPs is partly influenced by the strength of promoters controlling their expression [23]. Since the baculovirus polh is a stronger promoter than p10 [19], [23], [55], the cloning strategy of the ORFs coding structural rotavirus proteins was based on the number of copies required for each protein to form the viral capsid. ORFs of the proteins that requires more copies (VP6 = 260 trimers; VP7 = 260 trimers) were cloned downstream of the polh promoters, whereas those requiring fewer copies (VP2 = 60 dimers; VP4 = 60 trimers) were cloned downstream of the p10 promoters (Fig. 1). The ORFs for VP2 (C2 genotype) and VP6 (I2 genotype) from strain RVA/Human-wt/GR10924/1999/G9P[6], as well as VP4 and VP7 of various genotypes from selected study strains (Table 1) were cloned into a modified pFastBac vector, referred to as pFBq. Altogether, 17 recombinant baculoviruses were generated and were confirmed to express the proteins from the rotavirus ORFs that they carried. Fig. 2 and Fig. S2 shows selected examples of the expression of the various rotavirus proteins by these recombinant baculoviruses using SDS-PAGE and western blot. The various combinations of the baculoviruses that were generated are summarised in Table 2. Baculovirus titres ranging from 1×10^7^ to 1×10^9^ pfu/ml were determined using agarose-plaque assays. Recombinant VP2, VP4 and VP6 of approximately 100 kDa, 87 kDa and 45 kDa, respectively, were visualised on SDS-PAGE gels. Expression of recombinant VP7, of approximately 36 kDa, could only be confirmed with western blot (Fig. S2).

**Table 2:** A description of the pFastBACquad constructs prepared in this study from genomic data obtained from human stool samples containing human African rotavirus strains.

Cloning strategy	pFBq constructs
Monocistronic: with one ORF encoding VP7	pFBqG2^a^, pFBqG8^a^, pFBqG12^a^^,^ ¥
Monocistronic: with one ORF encoding VP4	pFBqP[4] b ^,^ ¥, pFBqP[8] b ^,^ ¥
Dualcistronic: with two ORFs encoding VP4 and VP7 or VP2 and VP6	pFBqG2P[6] c ^,^ ¥, pFBqG2P[4] c, pFBqG2P[8] c, pFBqG8P[4] d, pFBqG8P[8] d ^,^ ¥, pFBqG12P[4] e ^,^ ¥, pFBqG12P[8] e ^,^ ¥, pFBqG8P[6] f, pFBqG12P[6] f ^,^ ¥, pFBqG9P[4] f ^,^ ¥, pFBqG9P[8] f ^,^ ¥, pFBqG9P[6] f ^,^ ¥, pFBqC2I2 (VP2/VP6)

All codon-optimised ORFs were inserted into a commercial pUC57 transport plasmid at GeneArt (Life Technologies, New York, NY) and GenScript (GenScript USA Inc. New Jersey, NJ).

a **pFBqG2, pFBqG8 and pFBqG12**: Generated by ligating coding regions for G2, G8 and G12 VP7 proteins excised from pUC57G2, pUC57G2 and pUC57G8 plasmids to pFBq vector DNA, respectively. Both insert and vector DNA were prepared by double-digestion with *Bam* HI and *Not* I.

¥ VP4 and VP7 outer capsid proteins expressed by baculoviruses prepared from these expression cassettes were used to generate RV-VLPs in the current study. The VP2 and VP6 proteins that were prepared from strain RVA/Human-wt/ZAF/GR10924/1999/G9P[6] formed the scaffolds on to which the outer capsid proteins were assembled.

b **pFBqP** [4] **and pFBqP** [8]: Generated by ligating coding regions for P[4] and P[8] VP4 proteins excised from pUC57P[4] and pUC57P[8] plasmids to pFBq vector DNA. The vector and insert DNA were prepared by digestion with *Sma* I and *Spe* I.

c **pFBqG2P** [4] **, pFBqG2P** [8]: Generated by ligating the coding regions for P[4] and P[8] VP4 proteins excised from pUC57P[4] and pUC57P[8] plasmids to recombinant pFBqG2 expression cassettes, respectively. Both insert and vector DNA were prepared by double-digestion with *Sma* I and *Spe* I.

d **pFBqG8P** [4] **, pFBqG8P** [8]: Generated by ligating coding regions for P[4] and P[8] VP4 proteins excised with *Sma* I and *Spe* I from pUC57P[4] and pUC57P[8] plasmids to the recombinant pFBqG8 expression cassettes, respectively. The vector was also prepared by digesting with *Sma* I and *Spe* I.

e **pFBqG12P** [4] **, pFBqG12P** [8]: Generated by ligating the coding regions for P[4] and P[8] VP4 proteins excised from pUC57P[4] and pUC57P[8] plasmids to the recombinant pFBqG12 expression vector. Both the insert and vector were double-digested with *Sma* I and *Spe* I.

f **FBqG9P** [4] **, pFBqG9P** [8] **, pFBqG2P** [6] **, pFBqG8P** [6] **, pFBqG12P** [6]: Double-digestion of pFBqG9P[6] with *Sma* I and *Spe* I resulted in pFBqG9_*Sma*I/*Spe*I and P[6]_*Sma*I/*Spe*I DNA fragments. Double digesting pFBqG9P[6] with *Bam* HI and *Not* I resulted in pFBqP[6] *_Bam*HI/*Not*I and G9_*Bam*HI/*Not*I fragments. Recombinant pFBqG9P[4], pFBqG9P[8], pFBqG2P[6] expression cassettes were engineered by ligating coding regions for P[4] and P[8] VP4 proteins to the recombinant pFBqG9_*Sma*I/*Spe*I vector. Ligating G2_*Bam*HI/*Not*I, G8_*Bam*HI/*Not*I and G12_*Bam*HI/*Not*I fragments to recombinant pFBqP[6]_*Bam*HI/*Not*I vector resulted in pFBqG2P[6], pFBqG8P[6], pFBqG12P[6] expression cassettes, respectively.

### 3.2. Evaluation of the assembly of baculovirus-expressed rotavirus proteins into chimaeric rotavirus virus-like particles

To generate chimaeric RV-VLPs, the VP2/VP6 proteins were co-expressed with outer capsid proteins that comprised of various combinations of VP4 and VP7 consisting of G2, G8, G9 or G12 associated with either P[4], P[6] or P[8] genotypes. The recombinant baculovirus stocks were used to produce RV-VLPs by co-infecting insect cells with various infection strategies (Fig. 1). RV-VLPs were isolated from the supernatant of cultures harvested from both Sf9 and High Five insect cells co-infected with recombinant baculoviruses to determine if the yield could be improved with different cell lines as observed previously [35]. Two and three bands were usually visualised on sucrose gradients for dRV-VLPs and tRV-VLPs preparations, respectively.

#### 3.2.1. Verification of the presence of VP2, VP4, VP6 and VP7 on the assembled RV-VLPs using SDS-PAGE and western blot analysis

**Figure 3:**
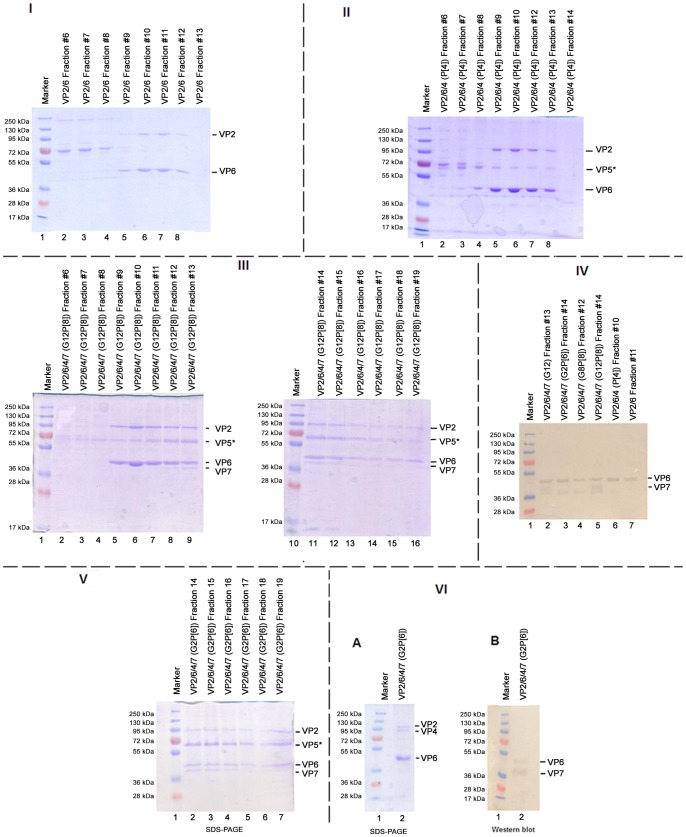
SDS-PAGE and western blot analysis of RV-VLP gradient fractions. Electrophoretic separation of selected gradient fractions, which contained structural proteins, out of the 18 fractions of 220 ul collected for each sample is depicted. (**I**) Gradient fractions for RV-VLPs (dRV-VLP) prepared with baculoviruses confirmed to express VP2 (C2 genotype) and VP6 (I2 genotype). (**II**) Gradient fractions for RV-VLPs (tRV-VLPs) prepared with baculoviruses confirmed to express VP2 (C2 genotype), VP4 (P[4] genotype) and VP6 (I2 genotype). (**III**) Gradient fractions for RV-VLPs (tRV-VLP) prepared with baculoviruses confirmed to express VP2 (C2 genotype), VP4 (P[8] genotype), VP6 (I2 genotype) and VP7 (G2 genotype). (**IV**) Detection of VP6 (I2 genotype) and VP7 (G8 genotype) with anti-rotavirus IgG antibodies using western blot in selected gradient fractions in which other structural proteins (VP2, VP5* or VP6) were detected using SDS-PAGE. (**V**) Gradient fractions for RV-VLPs on SDS-PAGE prepared by step-wise co-infection with baculoviruses confirmed to express VP2 (C2 genotype), VP4 (P[6] genotype), VP6 (I2 genotype) and VP7 (G2 genotype). (**VI**) Gradient fractions for RV-VLPs on SDS-PAGE (A) and nitrocellulose membrane, western blot (B) prepared by step-wise co-infection with baculoviruses confirmed to express VP2 (C2 genotype), VP4 (P[6] genotype), VP6 (I2 genotype) and VP7 (G2 genotype). An approximately 72 kDa non-specific band was consistently present in almost all sucrose gradient fractions #1 to #8 for each sample. The antibody used was weakly reactive against the inner VP2 capsid proteins in the RV-VLPs hence the absence of VP2 band in panel IV. Lane 1, Ladder, PageRuler Plus Prestain Protein Ladder (Fermentas UAB, Vilnius, Lithuania).

**Figure 4:**
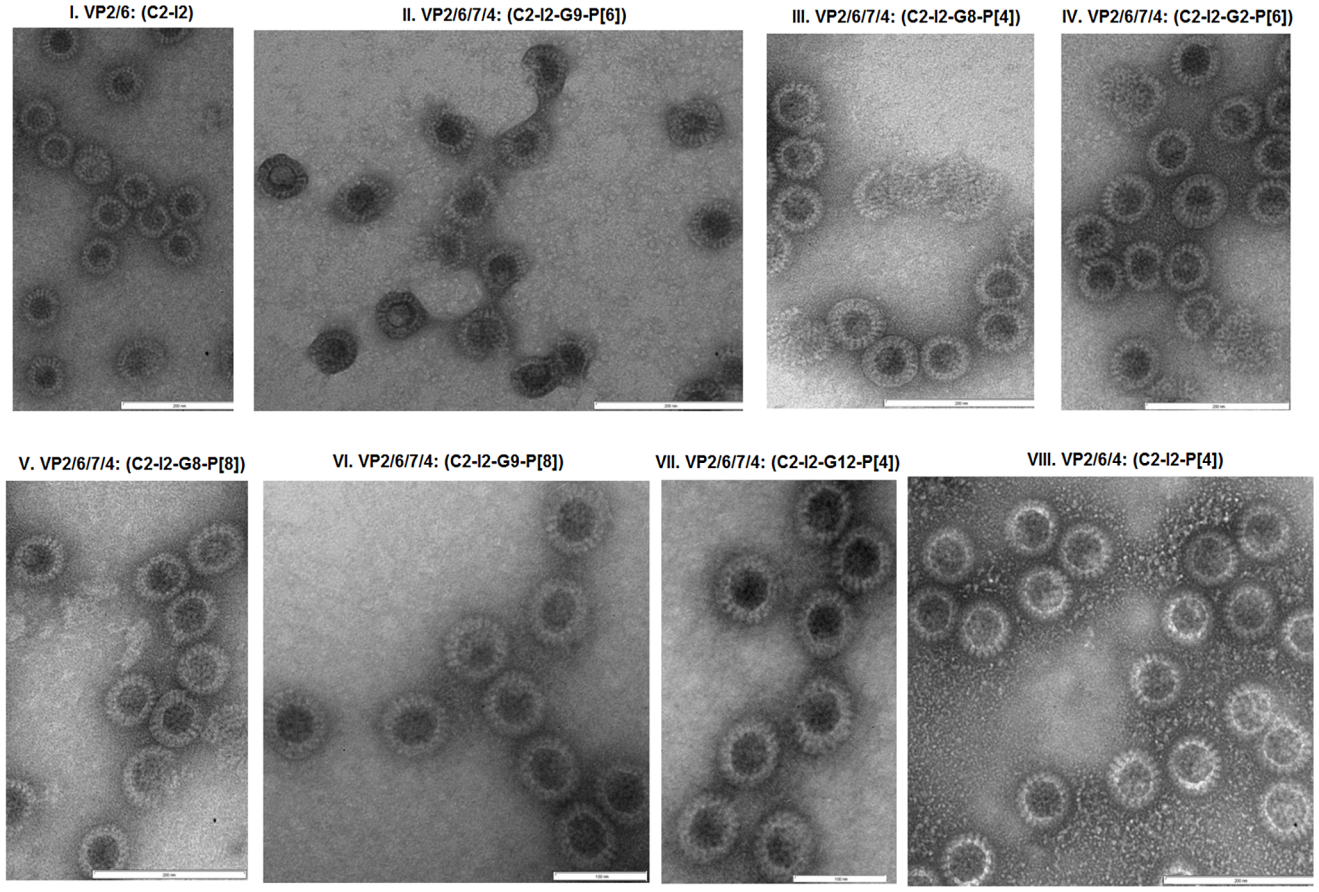
Rotavirus virus-like particles produced in insect cells by using dsRNA of wild-type strains. (**I**) dRV-VLPs produced by infecting High Five cells with recombinant baculoviruses containing ORFs coding for VP2 (C2) and VP6 (I2) proteins. Scale bar 200 nm. (**II**) tRV-VLPs produced by infecting Sf9 cells with recombinant baculoviruses containing ORFs coding for VP2 (C2), VP4 (P[6]), VP6 (I2) and VP7 (G9) proteins. Scale bar 200 nm. (**III**) Chimaeric tRV-VLPs produced by infecting High Five cells with recombinant baculoviruses containing ORFs coding for VP2 (C2), VP4 (P[4]), VP6 (I2) and VP7 (G8) proteins. Scale bar 200 nm. (**IV**) Chimaeric tRV-VLPs produced by infecting Sf9 cells with recombinant baculoviruses containing ORFs coding for VP2 (C2), VP4 (P[6]), VP6 (I2) and VP7 (G2) proteins. Scale bar 200 nm. **V**) Chimaeric tRV-VLPs produced by infecting High Five cells with recombinant baculoviruses containing ORFs coding for VP2 (C2), VP4 (P[8]), VP6 (I2) and VP7 (G8) proteins. Scale bar 200 nm. **VI**) Chimaeric tRV-VLPs produced by infecting High Five cells with recombinant baculoviruses containing ORFs coding for VP2 (C2), VP4 (P[8]), VP6 (I2) and VP7 (G9) proteins. Scale bar 100 nm. **VII**) Chimaeric tRV-VLPs produced by infecting Sf9 cells with recombinant baculoviruses containing ORFs coding for VP2 (C2), VP4 (P[4]), VP6 (I2) and VP7 (G12) proteins. Scale bar 100 nm. **VIII**) Chimaeric tRV-VLPs produced by infecting High Five cells with recombinant baculoviruses containing ORFs coding for VP2 (C2), VP4 (P[4]) and VP6 (I2) proteins. Scale bar 200 nm. tRV-VLPs with a smooth outer ring are shown with arrows.

Using SDS-PAGE, proteins VP2, VP4, VP6 and VP7 could be detected in fractions 9–15 of the sucrose gradients in most cases [Fig. 3 (I), (II), (III) and (V)], which correlated to the positions where the main band was located on the gradients. Often, VP4 appeared to have been cleaved by endogenous proteases since an approximately 60 kDa product was detected using SDS-PAGE which may represent a VP5* subunit, Fig. 3 (II lanes 5–8: where VP2 and VP6 were also visible; III lanes 5–15). A complete VP4 was only detected when tRV-VLPs were produced by mimicking assembly of infectious rotavirus particles [53] where insect cells were co-infected with baculoviruses expressing VP2/6 and VP4 first followed by step-wise infection 12 hpi with baculoviruses expressing VP7, Fig 3 (VI.A, lane 2). The fact that the commercial antibody used in the current study was raised against strain RVA/Cow-tc/USA/NCDV/1971/G6P6[1] which has a P[1] genotype could partially explain the difficulties experienced in detecting VP4 with western blot analysis from the RV-VLPs. Combining the total amount of RV-VLPs in the gradient fractions in which the recombinant structural rotavirus protein that constitute them were detected, concentrating them by centrifugation and determining the protein content, the average RV-VLP protein yield in High Five cells was almost three times higher than in Sf9 cells. Generally, for dRV-VLPs (VP2/6) an average 6.55 mg protein/L (approximately 8.33×10^16^ particles/L) and 1.99 mg protein/L (approximately 2.42×10^16^ particles/L) was recovered from 1×10^6^ of High Five and Sf9 cells, respectively. For tRV-VLPs (VP2/6/7/4), 1×10^6^ High Five and Sf9 cells respectively yielded 14.08 mg protein/L (approximately 9.23×10^16^ particles/L) and 5.75 mg protein/L (approximately 3.77×10^16^ particles/L), Fig. S3 and Table S1. Electron microscopy revealed that only approximately 10–30% of the tRV-VLPs were assembled into complete rotavirus particles, Fig. 4. Therefore, approximately 3.77×10^15^ to 1.13×10^16^ particles/L and 9.23×10^15^ to 2.77×10^16^ particles/L were assumed to be complete particles in Sf9 and High Five cells, respectively.

#### 3.2.2. Verification of RV-VLP assembly using transmission electron microscopy

On electron micrographs, dRV-VLPs were visible from gradient purified RV-VLPs produced through infection of insect cells with baculoviruses expressing VP2 and VP6, Fig. 4.I. tRV-VLPs were visualised from gradient purified RV-VLPs prepared by co-infecting insect cells with baculoviruses expressing VP2, VP4, VP6 and VP7. There was evidence of the formation of chimaeric tRV-VLPs that were produced by assembling outer capsid proteins made of various combinations of VP7 (G2, G8, G9 or G12) interchanged with VP4 (P[4], P[6] or P[8]) outer capsid onto G9P[6] dRV-VLPs (see Table 2 for the proteins expected to be expressed by the various generated recombinant baculoviruses). Representatives of the RV-VLPs produced in the current study are shown in Fig. 4. Up to 30% of the assembled RV-VLPs produced by homologous VP2/6/7/4 recombinant proteins derived from the same strain (RVA/Human-wt/ZAF/GR10924/1999/G9P[6]) were tRV-VLPs. The rest were dRV-VLPs without VP4 and VP7, Fig. 4 (II). When recombinant baculoviruses containing ORFs coding for VP7 with G2, G8, G12 genotypes or VP4 with P[4], P[6] or P[8] genotypes that were derived from heterologous rotavirus strains were used, the chimaeric tRV-VLP yield varied from 10–20%, Fig. 4 (III–VII). The distinction between the dRV-VLPs and tRV-VLPs was made based on the size and their morphology. dRV-VLPs were approximately 70 nm, whereas tRV-VLPs were approximately 75–80 nm. Furthermore, tRV-VLPs appeared to contain an extra smooth outer rim which may represent the VP7 layer, Fig. 4. II–VII [19]. However, this outer VP7 layer was partial in most of the tRV-VLPs that were obtained. Although the step-wise co-infection strategy resulted in the detection of complete VP4 when the RV-VLP sample were screened using SDS-PAGE, the amount and quality of the tRV-VLPs formed was not influenced by the strategy used.

## Discussion and Conclusion

In the current study, RV-VLPs were derived from the consensus insect cell codon-optimised sequence of rotavirus strains characterised directly from stool samples without prior adaptation to cell culture. dsRNA of rotavirus strains (G2, G8, G9 or G12 associated with either P[4], P[6] or P[8] genotypes) extracted directly from stool specimens were used. There are several reports of RV-VLPs preparations in insect cells, mammalian cells and larva using baculovirus expression systems [19], [56]–[59]. However, the majority have been derived from tissue culture adapted animal and human rotavirus strains. Adaptation of rotaviruses to tissue culture is labour-intensive, time consuming, requires personnel with specialized skills and multiple rounds of passage in primary cells [39], [60]. No study has yet attempted to prepare tRV-VLPs from dsRNA of rotaviruses extracted directly from clinical faecal specimens. Furthermore, most of the recombinant rotavirus proteins have previously been prepared from strains that have genotypes commonly associated with animal infections and very few recombinant rotavirus proteins bearing genotypes, other than those commonly characterised in humans such as G1 and G3 serotypes, have been produced [61], [62]. To date, expression of rotavirus proteins with G8, G9 and G12 serotypes/genotypes that emerged globally in the past two decades [49] which are also frequently characterised in African [49] and Asian countries [17] have not been reported.

Simultaneous expression of one or more recombinant rotavirus protein is required to produce RV-VLPs that mimic the structural conformation and size of the authentic native rotaviruses [19], thereby enabling easy uptake by dendritic cells. The large number of structural epitopes on RV-VLPs enables activation of B cells that subsequently leads to production of anti-rotavirus specific antibodies [63]. Therefore, RV-VLPs are potentially safe rotavirus vaccine candidates compared to live-attenuated candidates since RV-VLPs are devoid of genomic material. Furthermore, it has been proposed that broader protection might be achieved by preparing chimaeric RV-VLPs containing outer capsid proteins with different serotypes [19]. In the current study, attempts were made to prepare chimaeric tRV-VLPs by using dRV-VLPs (VP2/6) derived from strain GR10924 in combinations with VP4 and VP7 outer capsids from several strains listed in Table 1. The resultant chimaeric tRV-VLPs were designed to contain aVP2/bVP6/cVP4/dVP7 components where a = C2, b = I2, c  =  either P[4], P[6] or P[8] and d  =  either G2, G8, G9 or G12 genotypes of the proteins. Characterisation of the chimaeric tRV-VLPs that were prepared suggested that the outer rotavirus capsids of various combinations that were selected assembled onto the G9P[6] dRV-VLPs, (Fig. 4). Despite the correlation of the observed sizes and morphology of the tRV-VLPs generated in the current study to authentic RV-VLPs, the methods employed could not conclusively determine the assembly of VP4. Further cryo-electron microscopy and antigenicity characterisation would likely better visualise and detect the presence of VP4 spikes on the particles. Morphological studies could help to establish whether the RV-VLPs contain VP4 spikes, whereas serological or antigenicity studies would shed more light on whether VP4 spike conformation changes occurred due to co-expression of these proteins as has been observed in earlier classical reassortant RV-VLPs studies [64]. Of the RV-VLPs observed by TEM, about 30% were tRV-VLPs when VP2, VP4, VP6 and VP7 were derived from the same strain (GR10924). When the dRV-VLPs were co-expressed with outer capsid VP4 (with P[4], P[6] or P[8] genotypes) and VP7 (with G2, G8 or G12 genotypes) derived from heterologous strains, between 10 to 20% of the assembled particles were tRV-VLPs. The rest were dRV-VLPs. This may suggest that assembly of the outer capsid on the DS-1-like dRV-VLPs was not serotype-dependent, but could be improved by assembling recombinant structural VP2/6/7/4 derived from a homologous strain. Therefore, further optimization studies should be done to improve the assembly efficiency of the outer capsid layer onto the dRV-VLPs that may lead to production of stable complete homologous as well as chimaeric tRV-VLPs (VP2/6/7/4). The TOI and MOI are some of the parameters that will need to be optimised as several studies have shown their effect on the amount and quality on BEVS-produced RV-VLPs [65]–[67]. The current study further illustrated how the BEVS can be applied to generate tRV-VLPs containing different genotypes, albeit that the efficiency of their assembly will have to be improved in future investigations.

Based on the complexity of the structure of rotaviruses, wasteful accumulation of unassembled proteins or formation of incomplete particles can occur during assembly of RV-VLPs due to inadequate thermodynamics because of incorrect stoichiometric ratios of the recombinant structural proteins. Vieira and co-workers [23] demonstrated that only 15% of the expressed proteins correctly assemble into complete RV-VLPs. Incomplete RV-VLPs are often unstable [65] and result in the over-estimation of RV-VLP yields as isopycnic gradient centrifugation techniques, which are used as standard for quantification and purification, are unable to distinguish complete RV-VLPs from incomplete intermediaries or unassembled proteins [66]. Thus, although BVES is a useful and versatile system for recombinant protein expression which can be employed for producing vaccines for rapidly changing viruses, the process of producing RV-VLPs might be particularly challenging because: (i) RV-VLPs are complex structures that require assembly of several proteins simultaneously expressed in a single cell under various infection strategies; (ii) complex bioprocess manipulations are vital for efficient RV-VLPs production involving MOI, oxygen and nutrient requirements in culture media, reactor design for up-scaling and quantification of tRV-VLPs containing VP4; and (iii) downstream processes aimed at ensuring that RV-VLPs have correctly assembled and are devoid of contaminants are also complex [55]. Nevertheless, although highly specialized personnel are required to generate proof of concept of RV-VLP assembly, automated high-throughput production that requires little training have been amenable to large scale production successfully [63], [67]. In the present study, almost all baculoviruses with ORFs encoding VP2 and VP6 formed clearly defined wheel-like spherical dRV-VLPs that had rough surfaces in both Sf9 and High Five cells (Fig. 4.I) as observed by others [19], [22], which seem to suggest efficient RV-VLP assembly was achieved. Although the RV-VLP yield obtained in the present study was within the 0.5–13 mg protein/L range which has generally been reported by most research groups [68], yield under-estimation cannot be ruled out as only the fractions of sucrose gradients that correlated to the observed bands were used in quantification.

Different co-infection strategies were used to produce tRV-VLPs of which the step-wise approach appeared more likely to produce intact tRV-VLPs consisting of all four structural proteins (VP2/6/4/7) as intact 87 kDa VP4 and also an approximately 36 kDA VP7 band, similar to what Yao et al. [69] found, from the RV-VLPs was visible on most Coomasie blue stained SDS-PAGE gels (Fig. 3.V and VI). This observation correlates with the proposed assembly strategy of rotavirus protein during the replication process where VP4 is associated first with the DLP prior to the addition of the VP7 layer [70]. During natural rotavirus infection, VP4 is anchored by VP6 and protrudes through the five-fold apices of VP7 [29]. This suggests that assembling VP7 first may inhibit assembly of VP4 onto the dRV-VLP that attaches to VP6 as the VP7 channels may be blocked [53]. This may lead to formation of tRV-VLPs having a VP7 layer but devoid of VP4 spike proteins. Further cryo-EM studies of the RV-VLPs generated in the current study could shed more light on the order of assembly. Ciarlet et al. [71] concluded that the inclusion of VP7 and VP4 is not required to achieve protection from rotavirus infection in a rabbit model when Freund's adjuvant was used. Ciarlet and co-workers [71] reported high levels of protection ranging from 87 to 100% against rotavirus infection in individual rabbits immunised parenterally with VP2/6/7- or VP2/6-VLPs in Freund's adjuvant. This has also recently been supported by Zhou and co-workers [72] who achieved protection of up to 92% against rotavirus challenge in mice by administering dRV-VLPs with an adenovirus-expressed VP6 booster regime. Furthermore, the immune response against non-G1P[8] strains induced by the single G1P[8] strain Rotarix vaccine also points to heterotypic protection [5], [73]. Such findings warrant further investigation as to whether rationally designed genotype and serotype specific vaccines would be more effective against their target strains. Therefore, the dRV-VLPs prepared in the current study from wild-type rotavirus strains have the potential to be used as immunogens to formulate rotavirus vaccine candidates in future.

In conclusion, the current study proves the concept of generating chimaeric tRV-VLPs from rotavirus dsRNA extracted directly from clinical specimens. Assembly of VP7 outer capsids of various genotypes (G2, G8, G9 and G12) on a universal DS-1-like dRV-VLP (C2 and I2 genotypes) demonstrates the potential of using the BVES to tailor-made genotype specific RV-VLP vaccines for populations in targeted regions. The outcomes of the current study should support initiatives of developing alternative rotavirus sub-unit vaccines, especially in Africa where the burden of rotavirus diarrhoea is high. The novel approach of producing RV-VLPs using the consensus insect cell codon-optimised nucleotide sequence derived from whole-genome amplified cDNA [45]) which in turn was derived from dsRNA extracted directly from clinical specimens, introduced in this study, should speed up vaccine research and development by by-passing the need to adapt rotaviruses to tissue culture. Furthermore, the RV-VLPs produced with this approach would also mimic the infectious virus particle more closely as those produced from cell culture adapted strains since minimum changes would be introduced, for instance, in the epitope binding regions. Thus, it is now possible to generate RV-VLPs for evaluation as non-live vaccine candidates for any human or animal field rotavirus strain.

## Supporting Information

Figure S1 **Map of the pFastBACquad (pFBq) baculovirus transfer plasmid used for cloning and co-expression of rotavirus proteins in insect cells.** The elements of pFBq were derived from the pFastBac (Invitrogen, Life Technologies, Grand Island, NY) and pBACgus4x-1 (Novagen, Merck Chemicals Ltd., Nottingham, UK) transfer plasmids. The two polyhedron (polh) and two p10 promoters are located in the multiple cloning site (flanked by Tn7R and Tn7L transposition elements) that allow restriction enzyme-mediated cloning of foreign genes of interest.
(TIF)

Figure S2 **Evaluation of expression of VP7 by recombinant baculoviruses as indicated by SDS-PAGE (A) and duplicate western blot analysis (B).** (**I**) Recombinant VP7 expressed by five plaque-purified baculoviruses that contain VP7 encoding ORFs. (**II**) Recombinant VP4 and VP7 expressed by five plaque-purified baculoviruses that contain VP4 and VP7 encoding ORFs. (**III**) Recombinant VP7 (lane 2), VP4 (lane 3), VP4 and VP7 (lane 4), VP2 and VP6 (lane 5) expressed by amplified recombinant baculoviruses containing rotavirus ORFs encoding these proteins, respectively. Lanes 1. Ladder, PageRuler Plus Prestain Protein Ladder (Fermentas UAB, Vilnius, Lithuania). Wild-type, empty baculovirus used as a control.
(TIF)

Table S1 **Protein yield of RV-VLPs obtained from SF9 and High Five cells, and the approximated theoretical number of particles.**
(DOC)
