# Supplementary figures and images for: Chimaeric Virus-Like Particles Derived from Consensus Genome Sequences of Human Rotavirus Strains Co-Circulating in Africa
Source: PLoS One. 2014 Sep 30;9(9):e105167. doi: 10.1371/journal.pone.0105167 (PMC4181975; doi:10.1371/journal.pone.0105167)

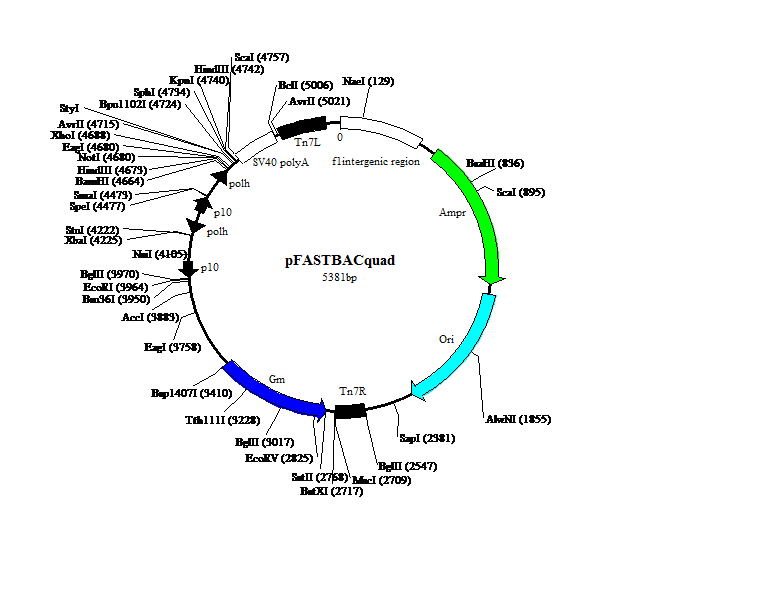

Supplement: Figure S1 — Map of the pFastBACquad (pFBq) baculovirus transfer plasmid used for cloning and co-expression of rotavirus proteins in insect cells. The elements of pFBq were derived from the pFastBac (Invitrogen, Life Technologies, Grand Island, NY) and pBACgus4x-1 (Novagen, Merck Chemicals Ltd., Nottingham, UK) transfer plasmids. The two polyhedron (polh) and two p10 promoters are located in the multiple cloning site (flanked by Tn7R and Tn7L transposition elements) that allow restriction enzyme-mediated cloning of foreign genes of interest. (TIF) [file pone.0105167.s001.tif]

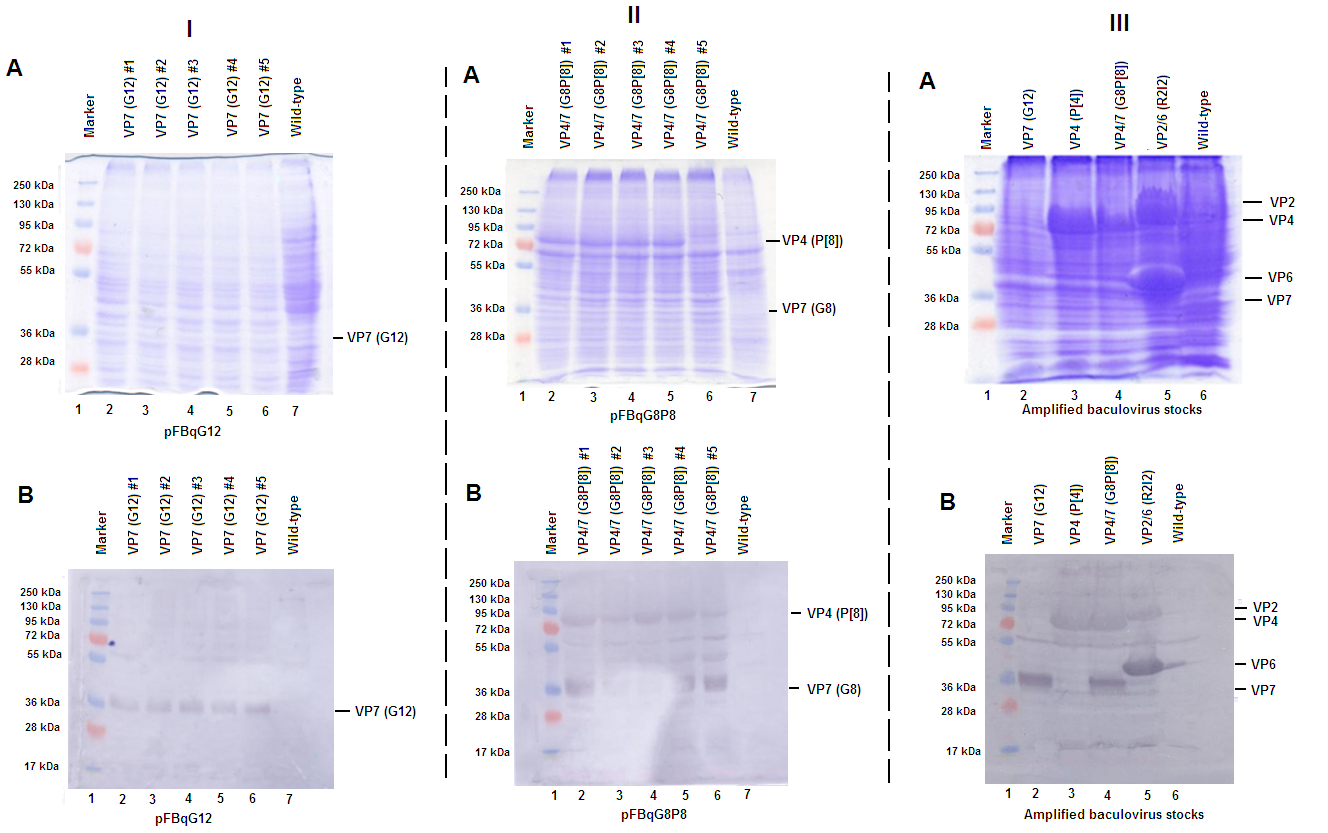

Supplement: Figure S2 — Evaluation of expression of VP7 by recombinant baculoviruses as indicated by SDS-PAGE (A) and duplicate western blot analysis (B). (I) Recombinant VP7 expressed by five plaque-purified baculoviruses that contain VP7 encoding ORFs. (II) Recombinant VP4 and VP7 expressed by five plaque-purified baculoviruses that contain VP4 and VP7 encoding ORFs. (III) Recombinant VP7 (lane 2), VP4 (lane 3), VP4 and VP7 (lane 4), VP2 and VP6 (lane 5) expressed by amplified recombinant baculoviruses containing rotavirus ORFs encoding these proteins, respectively. Lanes 1. Ladder, PageRuler Plus Prestain Protein Ladder (Fermentas UAB, Vilnius, Lithuania). Wild-type, empty baculovirus used as a control. (TIF) [file pone.0105167.s002.tif]
