# Supplementary material for: Chimaeric Virus-Like Particles Derived from Consensus Genome Sequences of Human Rotavirus Strains Co-Circulating in Africa
Source: PLoS One. 2014 Sep 30;9(9):e105167. doi: 10.1371/journal.pone.0105167 (PMC4181975; doi:10.1371/journal.pone.0105167)
Supplement: Table S1 — Protein yield of RV-VLPs obtained from SF9 and High Five cells, and the approximated theoretical number of particles. (DOC) [file pone.0105167.s003.doc]

**Table S1.** Protein yield of RV-VLPs obtained from SF9 and High Five® cells and the approximated theoretical number of particles

|  | **DLP (VP2 + VP6)** | | **TLP (VP2 + VP6 + VP4 + VP7)** | | |
| --- | --- | --- | --- | --- | --- |
|  | **^1^Yield**  **(mg protein/L)** | **Number of particles/L** | **^1^Yield**  **(mg protein/L)** | ^2^**Number of particles/L (total)** | ^2^**Number of particles/L (complete; 10 - 30%)** |
| **Sf9** | 1.99 | 2.42 x 10^16^ | 5.75 | 3.77 x 10^16^ | 3. 77 x 10^15^  - 1.13 x 10^16^ |
| **High Five®** | 6.55 | 8.33 x 10^16^ | 14.08 | 9.23 x 10^16^ | 9.23 x 10^15^ - 2.77 x 10^16^ |

^1^ Protein yield after recovery of RV-VLPs/1 x 10^6^ cells by sucrose gradient. The concentration values, determined with the Bicinchoninic Acid (BCA) Protein Assay Reagent (Pierce, Rockford, IL), for RV-VLPs indicated in the table are averages obtained from baculoviruses expressing rotavirus proteins with G9, G8, G12, P[4], P[6] and P[8] genotypes. The RV-VLPs were produced in 100 ml shaker cultures and the listed yields were converted from µg/100 ml to mg/L.

^2^ The following assumptions were made to calculate the number of particles per yield based on the number of size of the constituent proteins for RV-VLPs and the number of molecules making up each capsid per virion:

DLP weight = 47340 kDa (VP2: [102 *120 kDa] + VP6: [45 * 780 kDa]);

TLP weight = 91860 kDa (VP2: [102 *120 kDa] + VP6: [45 * 780 kDa]) + VP7: [37 *780 kDa] + VP4: [87 * 180 kDa]).

1 kilogram = 6.02213665168 x 10^26^ dalton ([www.convertunits.com](http://www.convertunits.com/)).

The calculation for particle numbers:

1. **For VLP in Sf9 cells:**

Single particle weight = 47340 kDa

Since: 1 dalton = 1.6605402 x10^-24^ grams

1 kDa= 1.6605402 x10^-21^ grams

Therefore, 1 DLP weighs:

47340 kDa x 1.6605402 x10^-21^ = 7.860997306799999 ^-20^ grams

Since total RV-VLP yield is 1.99 mg/L = 0.0019g/L

To find the number of particles per L, total mass/weight of a single particle:

0.0019g/L

7.860997306799999 ^-20^

**= 2.42 x 10^16^ particles/L**

1. **For tRV-VLP in High Five cells:**

Particle weight = 91860 kDa

Since: 1 dalton = 1.6605402 x10^-24^ grams

1 kDa = 1.6605402 x10^-21^ grams

Therefore, 1 TLP weighs:

91860 kDa x 1.6605402 x10^-21^ = 1.52537222772 ^-19^ grams

Since total RV-VLP yield is 14.08 mg/L = 0.01408g/L

To find the number of particles per L, total mass/weight of a single particle:

0.01408g/L

1.52537222772 ^-19^

**= 9.23 x 10^16^ particles/L**

**Since 10 – 30% of the RV-VLP were assumed to be TLP; approximately 10 – 30% of the total amount was 9.23 x 10^15^ - 2.77 x 10^16^**
